# Insulin Resistance, Hyperinsulinemia and Atherosclerosis: Insights into Pathophysiological Aspects and Future Therapeutic Prospects

**DOI:** 10.2174/011573403X314035241006185109

**Published:** 2024-10-16

**Authors:** Georgios S. Papaetis, Anastasia Sacharidou, Ioannis C. Michaelides, Konstantinos C. Mikellidis, Stylianos A. Karvounaris

**Affiliations:** 1 K.M.P Therapis Paphos Medical Center, Internal Medicine and Diabetes Clinic, 14 Vasileos Georgiou B Street, Office 201, 8010, Paphos, Cyprus;; 2 CDA College, 73 Democratias Avenue, Paphos, Cyprus;; 3 K.M.P Therapis Paphos Medical Center, Cardiology Clinic, 14 Vasileos Georgiou B Street, Office 201, 8010, Paphos, Cyprus;; 4 K.M.P Therapis Paphos Medical Center, Obstetrics and Gynecology Clinic, 14 Vasileos Georgiou B Street, Office 201, 8010, Paphos, Cyprus

**Keywords:** Insulin, insulin resistance, hyperinsulinemia, atherosclerosis, cardiovascular disease, glucose

## Abstract

Insulin resistance describes the lack of activity of a known quantity of insulin (exogenous or endogenous) to promote the uptake of glucose and its utilization in an individual, as much as it does in metabolically normal individuals. On the cellular level, it suggests insufficient power of the insulin pathway (from the insulin receptor downstream to its final substrates) that is essential for multiple mitogenic and metabolic aspects of cellular homeostasis. Atherosclerosis is a slow, complex, and multifactorial pathobiological process in medium to large arteries and involves several tissues and cell types (immune, vascular, and metabolic cells). Inflammatory responses and immunoregulation are key players in its development and progression. This paper examines the possible pathophysiological mechanisms that govern the connection of insulin resistance, hyperinsulinemia, and the closely associated cardiometabolic syndrome with atherosclerosis, after exploring thoroughly both *in vitro* and *in vivo* (preclinical and clinical) evidence. It also discusses the importance of visualizing and developing novel therapeutic strategies and targets for treatment, to face this metabolic state through its genesis.

## 1 INTRODUCTION

Insulin resistance describes the lack of activity of a known quantity of insulin (exogenous or endogenous) to promote glucose uptake and its utilization in an individual, as much as it does in metabolically normal individuals [1]. On the cellular level, it suggests insufficient power of the pathway of insulin (starting from the insulin receptor cascade to its terminating substrates) that is essential for multiple mitogenic and metabolic aspects of cellular homeostasis [1, 2]. IR is manifested not only by less insulin activity but also by a slow-going action of insulin to trigger glucose utilization [3]. Several mechanisms are responsible for its development. These mainly include (i) fetal malnutrition; and (ii) acquired situations that suppress the activity of insulin to its target cells. These are mainly increased visceral adipose tissue, glucotoxicity, lipotoxicity, and age; and (iii) genetic defects; these include abnormalities of insulin and one or more molecules related to the insulin cascade,auto-antibodies blocking insulin and/or the insulin receptor, accelerated insulin metabolism and abnormal mitochondrial function [1-6]. In response to an IR state, pancreatic beta (β) cells produce higher quantities of insulin to compensate. Hence, normal glucose tolerance (NGT) is established. In any other case, euglycemia cannot be achieved. Gradual β-cell degeneration and loss are associated with the evolution from NGT to impaired glucose tolerance (IGT) and finally type 2 diabetes (T2D) [7].

## 2 ATHEROSCLEROSIS

Atherosclerosis is a slow, complex, and multifactorial pathobiological process in medium to large arteries and involves several tissues and cell types (immune, vascular, and metabolic cells) [8]. In brief, endothelial dysfunction and the subsequent loss of their ability to maintain vascular homeostasis is the crucial first step in the initiation of this process. Low-density lipoprotein cholesterol (LDL-C) particles are trapped in the subendothelial space and oxidized [8]. LDL-C retention and their modification in addition to several other atherogenic factors promote monocyte attachment into the intima. Altered LDL-C is eventually trapped by differentiated monocytes, as well as by vascular smooth muscle cells (VSMCs) and endothelial cells (ECs). The deposition of esterified and free cholesterol in these cells promotes the formation of foam cells [8, 9]. Furthermore, several molecular and cellular inflammatory cascades are stimulated, promoting fatty streak development (the first sign of atherosclerosis), which is the result of significant accumulation of lipids inside the cells and the extracellular matrix [10]. As atherosclerotic plaque evolves, the necrotic core (the nucleus of the plaque) becomes bigger, mainly because of macrophage death and impaired efferocytosis. The stabilization of the necrotic core is achieved after it is gradually filled by fibers, thus establishing a fibrous cap [11]. The fibrous cap is a subendothelial border between the lumen of the vessel and the core with necrosis. It consists of extracellular material secreted from VSMCs. Its role is to confer structural support and prevent prothrombotic molecules of the core from being exposed and eventually trigger thrombosis. Both the necrotic core and the fibrous cap are considered as the contents of advanced atherosclerosis. Calcification of atheroma plaques are bone-like formations inside the plaques. Calcium orthophosphate penetrates the nucleation sites, which are transformed into amorphous calcium phosphate crystals and eventually to crystalline formations [8, 11, 12].

## 3 IR AND ATHEROSCLEROSIS

### 3.1 Introduction

The concept that excessive insulin levels could contribute to the development of atherosclerosis was published in 1969 from Stout *et al* [13]. The first evidence for a direct association of IR *per se* with atherosclerosis with no symptoms (in femoral or carotid arteries) was published by Laakso *et al* in 1991, after applying the *hyperinsulinemic-euglycemic* clamp (HIEC) procedure in 30 nondiabetic nonobese individuals [14]. In 1996 Bressler *et al*. described for the first time after using the HIEC procedure in 13 healthy, normotensive, NGT participants with angiographically documented severe coronary artery disease (CAD), that they were significantly IR *versus* well-matched NGT individuals without CAD; positive association was also found between the severity of CAD and IR [15]. During the same year a published sub-analysis of the Quebec Cardiovascular Study, in which participants with diabetes were excluded from the matched investigation, suggested that men who experienced CAD had baseline fasting insulin levels 18% higher *versus* those without CAD (*p<*0.001); higher insulin levels were independently related to increasing risk of CAD [odds ratio (OR) for CAD with every increase of one standard deviation (SD) of insulin levels was 1.7; 95% CI: 1.3-2.^4^] [16]. Since then, mounting epidemiological evidence has demonstrated that IR and/or hyperinsulinemia were powerful predictors of atherosclerotic cardiovascular disease (ASCVD). In 2012 a large meta-analysis, which investigated data from 65 studies (involving approximately half a million individuals without diabetes) showed that the homeostatic model assessment of insulin resistance (HOMA-IR) was related to 64% higher risk for CAD, 76% for stroke (ischaemic or haemorrhagic) and 44% for CVD comparing high to low values [17]. Interestingly, the risk for CAD was higher by 46% for an increase of one SD for HOMA-IR.

Furthermore, the pivotal Diabetes Control and Complication Trial (DCCT) has shown that patients with the highest baseline levels of IR [as assessed by a low glucose disposal rate (eGDR)] were exposed to the biggest subsequent danger of experiencing macrovascular complications of type 1 diabetes (T1D); this association was independent of their treatment arm and their total insulin dose [18]. IR *per se* has been also related to a higher risk for atherosclerosis (clinical or subclinical) in individuals with T2D and individuals with other IR-associated disorders beyond T2D such as prediabetes (especially IGT), non-alcoholic fatty liver disease (NAFLD)/non-alcoholic steatohepatitis (NASH), gestational diabetes (GDM), polycystic ovary syndrome (PCOS) and obstructive sleep apnoea (OSA) [4,19-24]. Interestingly, the investigation of a large Korean population-based cross-sectional database suggested that lean highly IR individuals with NAFLD had significantly higher scores for ASCVD and a higher probability of experiencing ASCVD *versus* obese IR individuals with NAFLD [25]. This manuscript explores the possible pathophysiological mechanisms that govern the association of atherosclerosis with IR, hyperinsulinemia, and the closely associated cardiometabolic syndrome, after exploring thoroughly both *in vitro* and *in vivo* (preclinical and clinical) evidence. It also discusses future therapeutic prospects to face IR through its genesis.

### 3.2 The Insulin Pathway

**Fig. (1):**
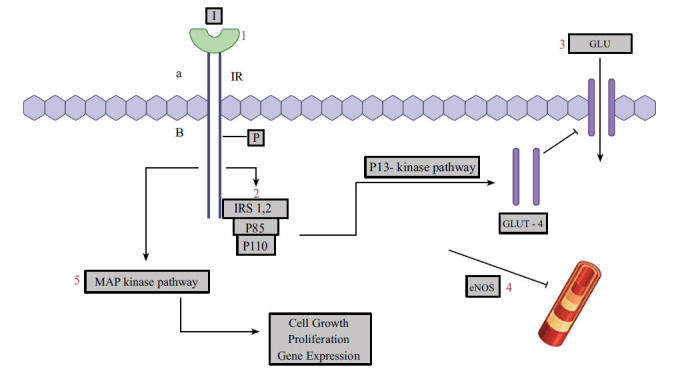
**1.** Insulin exerts its activity after binding to the α-subunit of the insulin receptor at the extracellular surface of the sarcolemmal membrane promoting conformational alterations **2.** This, in turn, causes autophosphorylation of the β-subunit of the insulin receptor and a subsequent tyrosine phosphorylation of intracellular adapter proteins, such as IRS-1 and IRS. The interaction between the p85 regulatory subunit of PI 3-kinase and the phosphorylated IRS stimulates the p110 catalytic subunit of PI 3-kinase **3.** Stimulation of p110 subunit activates the production of PIP3 from PIP2 **4.** PIP3 eventually recruits PDK1, which phosphorylates and activates Akt (also known as PKB). The activation Akt/PKB finally promotes the phosphorylation of several downstream substrates in major metabolic tissues (such as liver, skeletal muscle and adipose tissue) **5.** Specifically in skeletal muscle cells the insulin pathway is associated with glucose transport, through stimulation and translocation of GLUT-4 to the cell surface **6.** Activation of PI 3-kinase pathway promotes the phosphorylation of eNOS and stimulates its expression and activity. Eventually it increases NO levels that stimulate smooth muscle relaxation and vasodilation **7.** Tyrosine-phosphorylated Shc and IRS proteins can also lead to the activation of MAP kinase signalling pathway, which is associated with cell growth, proliferation and multiple gene expression. IRS: Insulin receptor substrate; PI 3-kinase: Phosphatidylinositol 3-kinase; PKB: Protein kinase B; GLUT-4: PIP3: Phosphatidylinositol-3,4,5-trisphosphate; PIP2: Phosphatidylinositol-4,5-bisphosphate; PDK1: 3-phosphoinositide-dependent kinase-1; Glucose transporter 4; eNOS: Endothelial nitric oxide synthase; MAP: Mitogen-activated protein; I: Insulin; GLU: Glucose.

Insulin receptor is a transmembrane protein that consists of four subunits: two alpha (α) and two β. The activity of insulin in nonvascular cells is promoted after bonding to its α-subunit at the extracellular area of the sarcolemmal membrane promoting conformational alterations [26]. This in turn, stimulates the autophosphorylation of the intracellular area inside the β-subunit of the insulin receptor, which has a tyrosine kinase (TK) effect [26, 27]. Three tyrosine residues (Tyr^1163^, Tyr^1162,^ and Tyr^1158^) should be phosphorylated to amplify TK activity [27]. Eventually tyrosine phosphorylations of adapter proteins intracellularly [such as insulin receptor substrates 1 and 2 (IRS-1 and IRS-2), growth factor receptor-bound protein-2 (GRB-2), GRB-10, and several Shc proteins] are triggered, which attract multiple intracellular signalling intermediate molecules [28-30]. In myocytes, IRS-1 is the major docking protein, while in hepatocytes IRS-2 phosphorylation mainly promotes the effects of insulin [28, 29]. Tyrosine-phosphorylated IRS-1/2 and Shc proteins become targets of attachment for several intracellular proteins that contain Src-homology 2 (SH2) and are responsible for the variety of the biological effects of insulin [29-31]. Two major pathways are eventually activated: (i) the phosphatidylinositol 3-kinase (PI 3-kinase) pathway; and (ii) the extracellular signal-regulated kinase (ERK)/mitogen-activated protein kinase (MAPK) pathway. The PI 3-kinase pathway is recruited after the phosphorylation of IRS. The interaction between the p85 regulatory subunit of PI 3-kinase and the phosphorylated IRS stimulates the p110 catalytic subunit of PI 3-kinase, which eventually promotes its activation. The PI 3-kinase pathway stimulates important downstream substrates in major human cells (such as hepatocytes, skeletal myocytes, and adipocytes) (Fig. **1**) [29-31].

#### 3.2.1 Skeletal Myocytes

Specifically in skeletal myocytes the insulin pathway is associated with: (i) transportation of glucose, through the expression and translocation of glucose transporter 4 (GLUT-4) to the cell membrane. During studies using the HIEC technique, it was demonstrated that about 80% of all glucose uptake from the human body is found in skeletal muscles. Transportation of glucose is the rate-controlling effect of insulin, to achieve its cellular effects and a very early event in the pathogenesis of T2D; (ii) phosphorylation of glucose through Hexokinase-II expression; (iii) glycogen synthesis through the activation of glycogen synthase (GYS). It also controls glycogen phosphorylase activity through dephosphorylation of phosphorylase kinase; and (iv) stimulation of mechanistic target of rapamycin complex 1 (mTORC1), which suppresses autophagy and regulates protein synthesis and cell growth (by activating ribosome biogenesis and mRNA translation through phosphorylation of downstream molecules). Higher mTORC1 secretion promotes higher protein synthesis and deterioration of several cellular activities, mostly due to decreased cellular autophagy. P70S6 kinase (S6K1 kinase) also has an essential role in protein synthesis [31-33].

#### 3.2.2 Hepatic Cells

Approximately 30% of ingested glucose after a meal is taken by the liver. In the liver, the PI3-kinase/Akt pathway: (i) suppresses the production of glucose, namely glycogenolysis and gluconeogenesis. The suppression of gluconeogenesis is achieved by inhibiting lipolysis from the adipose tissue (thus reducing glycerol substrates) and by Akt-induced phosphorylation of forkhead box O1 (FOXO1) through IRS-2 at Thr^24^, Ser^253^ and Ser^316^ (promoting its exclusion from the nucleus and preventing the transcriptional expression of gluconeogenic genes); (ii) enhances glycogen synthesis after the regulation of GYS and glycogen phosphorylase; and (iii) stimulates *de novo* lipid anabolism by activating its key transcriptional molecule sterol regulatory element-binding protein 1c (SREBP-1c) through IRS-1 [34-38].

#### 3.2.3 Adipocytes

In adipocytes insulin: (i) downregulates lipolysis, which in turn suppresses gluconeogenesis through decreased gluconeogenic substrates. This effect is mainly achieved by attenuating c-AMP stimulated adrenergic activities (such as hormone-sensitive lipase phosphorylation and perilipin phosphorylation) through phosphodiesterase 3B (PDE3B) activation; and (ii) promotes lipogenesis after (1) regulating fatty acid transport proteins (FATPs) and non-esterified fatty acids (NEFAs) esterification; (2) by activating SREBP-1c; and (3) by regulating the generation of peroxisome proliferator-activated receptor-gamma (PPAR-γ) in human adipose tissue [39-42].

#### 3.2.4 VSMCs and ECs

VSMCs and ECs also express insulin receptors [43]. Phosphorylation of insulin receptors promotes tyrosine phosphorylation of IRS-1, IRS-2, and Shc. Hence, both PI 3-kinase and ERK/MAPK pathways are stimulated in normal metabolic conditions. Furthermore, after the activation of the PI 3-kinase/Akt pathway (mainly through IRS-1), insulin triggers the phosphorylation of endothelial nitric oxide synthase (eNOS) at Ser^1177,1179^ and stimulates its expression and activity. Eventually, it increases NO levels that stimulate smooth muscle relaxation and vasodilation [41, 42]. PI 3K inhibitors can blunt NO production [44]. 5' adenosine monophosphate-activated protein kinase (AMPK) is activated from several stimuli (including adiponectin) and can stimulate the PI 3-kinase cascade; AMPK also suppresses the expression of nuclear factor-κB (NF-κB) and NADPH oxidase 4 (NOX4) overactivity, which promote high oxidative stress.

#### 3.2.5 Monocytes/macrophages and T lymphocytes

Insulin signaling is also activated in monocytes/macrophages, although IRS-1 isoform is indetectable [45]. Suppression of the insulin pathway in monocytes was shown to decrease the protective activity of insulin to suppress macrophage apoptosis; this effect was achieved mainly due to lower expression of the anti-apoptotic gene *Bcl-x* [46]. T lymphocytes do not have insulin receptors but possess atypical capability to express them in the presence of an antigen *in vivo* [43].

#### 3.2.6 Other Cells

In renal cells, insulin has significant effects on the modulation of gluconeogenesis and lipolysis. Specifically, in glomerular podocytes, it stimulates glucose transport and in tubular cells it promotes glucose reabsorption, and regulation of gluconeogenesis contributing to sodium homeostasis [47]. In cardiomyocytes, activation of the insulin cascade controls diverse cellular events ranging from cell growth, differentiation, and cell viability to suppression of apoptosis and autophagy [48]. Activation of insulin signaling in brain cells controls nutrient homeostasis, cognition, and several important neurotrophic and neuromodulatory effects (Table **1**) [49].

### 3.3 PI 3-kinase/Akt Pathway and IR

A wealth of evidence, both in preclinical models and in individuals with IR, has demonstrated that the TK activity of insulin receptors and/or the number of insulin receptors are either normal or only moderately downregulated (secondary due to chronic hyperinsulinemia). However, this evidence was insufficient to justify the significant reduction of insulin activity [44]. Other possible mechanisms described were the overexpression of p85α of PI 3-kinase (a major regulatory subunit) and the subsequent imbalance among the PI 3-kinase subunits leading to lower affinity of PI-3 kinase with IRS-1/2 [44, 50]. However, most of the well-established evidence indicates that the molecular mechanisms of IR are defects in the post-receptor level and specifically through multi-site serine/threonine phosphorylation of IRS proteins (more than 70 potential serine sites have been described on IRS-1) [6, 51]. Excessive serine/threonine phosphorylation (in contrast to tyrosine phosphorylation) of IRS proteins can promote dissociation between the insulin receptor with IRS-1/2 and/or suppression of the effect of IRS to attract PI 3-kinase, thus preventing its stimulation. Stimulated serine/threonine phosphorylation can also accelerate proteasomal degradation of IRS [52, 53].

#### 3.3.1 Molecular Mechanisms

Several molecules can promote serine/threonine IRS phosphorylation [4, 50, 54-66] (Table **2**):

(i) Adipocytokines [such as tumor necrosis factor α (TNF-α), interleukin 1 (IL-1), ΙL-6, resistin, angiotensinogen, *m*onocyte *c*hemoattractant *p*rotein-1 (MCP-1/CCL2)].

(ii) m-TOR/S6K1 kinase activation during hyperinsulinemia and overnutrition.

(iii) Stimulation of c-Jun N-terminal kinase (JNK) and IkB kinase (IKK) β (IKKβ)/NF-kB pathways after the arrival of several extracellular signals (such as adipocytokines, lipid intermediates, and high oxidative stress).

(iv) Defective stimulation of protein kinase C (PKC) isoforms.

(v) During an IR sate the activity of insulin to decrease lipolysis from adipocytes (chiefly in visceral hypertrophic adipocytes) is significantly reduced, even during times of nutrient excess. Hence, higher amounts of NEFAs are delivered both in muscle and hepatic cells. Moreover, mitochondrial oxidative phosphorylation is suppressed (because of mitochondrial dysfunction and/or reduced mitochondrial content) and β-oxidation is downregulated. When the delivery rate of NEFAs to the liver and muscle exceeds the rate of intracellular β-oxidation and/or conversion to neutral triglycerides (TG), excess NEFAs can enter into harmful non-oxidative pathways. Hence they promote the accumulation of lipid intermediate metabolites [lysophosphatidic acid (LPA), diacylglycerol (DAG), acylcarnitines, and ceramides]. Defects of the insulin signalling cascade from higher intracellular concentrations of these intermediate molecules have been shown in various studies and largely: (1) *DAG.* DAG is a key molecule in this setting and inhibits the insulin cascade by promoting IRS serine/threonine phosphorylation. These activities are achieved through (A) PKC either by itself (in liver cells PKCε promotes the phosphorylation at Thr^1160^ of IRS-2 and in myocytes, PKC_θ_ stimulates the phosphorylation of IRS-1 at Ser^1101^ or (B) through several pro-inflammatory signalling cascades (such as JNK, IKKβ/NF-kB and mTOR); (2) *Ceramides.* They can suppress the stimulation of the PI-3 kinase/Akt pathway through increased activity of protein phosphatase-2A that promotes dephosphorylation and deactivation of Akt. They also impair Akt translocation to the plasma membrane (mediated by PKC-ζ activation); and (3) *Fatty acyl-CoA derivatives.* Elevated NEFAs are also converted to their fatty Acyl-CoA derivatives, which stimulate the IKKβ/NF-kB signalling cascade and promote the production of major stimulating key-inflammatory mediators (TNFα, ILs, and PKC), which are required for atherosclerotic plaque formation, evolution, and destabilization.

(vi) Endoplasmic reticulum (ER) stress can induce IR in hepatocytes and the pancreatic β-cells either directly or indirectly through several inflammatory signalling pathways. It can also stimulate toll-like receptor 4 (TLR-4) promoting IKKβ/NF-kB activation and eventually atherogenesis.

(vii) Reactive species, mainly reactive oxygen species [ROS: superoxide anion, hydrogen peroxide (H_2_O_2_), and hydroxyl radical ions] are found at low physiological levels mostly in peroxisomes and mitochondria. During an IR state excessively high levels of ROS are described due to: (1) increased NEFAs oxidation leads to overabundance of reflexed protons in the electron transport chain, which in turn promote altered electron transport kinetics and stimulation of escape electron accepting pathways (such as reaction with oxygen to form superoxide); (2) PI3-kinase can induce the phosphorylation of Rac instead of PIP2 and can stimulate the expression and activity of NOX subunits 1,2 and 4. NOX-4 is a strong oxidizing enzyme that stimulates ROS production. High ROS levels can directly stimulate several inflammatory pathways (mainly JNK and MAPK p38) resulting in mitochondrial-induced stress responses. They can also promote IR by affecting several important proteins of the insulin cascade (such as IRS-1 phosphorylation/redistribution). ROS also contributes to vascular dysfunction and the evolution of a proatherogenic state, especially in patients who experience higher markers of genomic unsteadiness and oxidative DNA defects in peripheral blood mononuclear cells.

(viii) Stimulation of angiotensin II (Ang II) can induce IR through ROS-mediated serine phosphorylation of IRS-1 in VSMCs, skeletal myocytes, and through JNK/ERK 1/2 serine phosphorylation of IRS-1 in the ECs. Stimulation of mineralocorticoid receptors also activates the mTOR/S6K1 kinase signaling pathway that promotes IRS-1 serine phosphorylation in adipocytes, hepatic cells, skeletal myocytes, and cardiac cells. Interestingly, high insulin levels can stimulate the effect of Ang II to transactivate NF-kB leading to inflammation, hypertension, and atherosclerosis. Furthermore, both insulin and Ang II can stimulate the effect of glucocorticoid kinase 1 (SGK-1) in ECs which is a key modifier of vascular and kidney sodium channel function; increased sodium flux promotes remodelling of the cytoskeleton and vascular stiffening.

### 3.4 Suppression of Nitric Oxide Pathway and Atherosclerosis

Of major significance in keeping vascular homeostasis is the secretion of NO. NO stimulates smooth muscle relaxation and vasodilation. It has also essential anti-thrombogenic and anti-inflammatory properties [67]. It suppresses VSMC proliferation, leukocyte adhesion/migration, as well as platelet activation and adhesion [67, 68]. Insulin stimulates PI 3-kinase/Akt (mainly at IRS-1) and through this pathway, it promotes the phosphorylation of eNOS at serine^1177,1179^. In this way, it enhances eNOS expression/activity and eventually increases NO levels [69].

Downregulation of the PI 3-kinase pathway in vascular cells suppresses the vasodilator activity of insulin through NO production. In this way, it promotes high blood pressure (BP) and endothelial dysfunction. Furthermore during an IR state, suppressed NO levels are associated with (i) proliferation and migration of VSMCs since insulin can no longer maintain a differentiated phenotype of VSMCs or antagonize the proliferative action of *platelet-derived growth factor* (PDGF); (ii) loss of the protective activity of NO in the endothelium regarding the detrimental activities of *vascular endothelial growth factor* (VEGF) on the production of adhesion molecules; interestingly, compensatory hyperinsulinemia enhances the interplay of ECs with circulating monocytes, promoting this very first step in the pathogenesis of atherosclerotic disease; (iii) oxidative modification of lipoproteins and phospholipids; and (iv) platelet aggregation [67-70]. It should be also emphasized that the endothelium is significantly involved in the transport of insulin from the vasculature to skeletal muscle cells, as insulin undergoes transcytosis through the endothelium; this process is rate limiting for the activity of insulin and is stimulated from eNOS [71]. During an inflammatory state, C-reactive protein (CRP) was shown to activate endothelial Fcg receptor IIB (FcgRIIB) and eventually inhibit eNOS activation. Hence it blocks skeletal muscle insulin delivery and promotes IR [72].

### 3.5 Stimulation of ERK/MAPK Signalling Pathway and Atherosclerosis

**Fig. (2):**
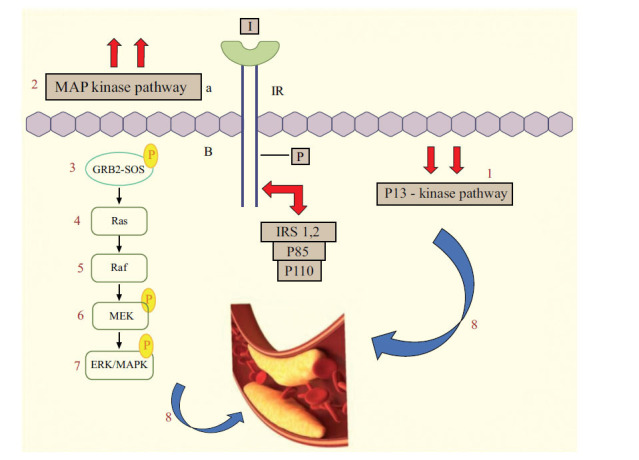
In patients with elevated IR, cellular events that lead to the activation of PI-3 kinase pathway are downregulated (selective IR). Excessive serine/threonine phosphorylation (in contrast to tyrosine phosphorylation) of IRS proteins can lead to dissociation between the insulin receptor with IRS-1/2 and/or suppression of IRS ability to attract PI 3-kinase. Downregulation of PI 3-kinase pathway in vascular cells suppresses the vasodilator activity of insulin through NO production. In this way it promotes high blood pressure, endothelial dysfunction, oxidative modification of lipoproteins/phospholipids and platelet aggregation 2. On the contrary, the MAP kinase signalling pathway stays insulin sensitive. Hence, it is excessively hyperactive and promotes the growth and atherogenic effects of insulin 3. Specifically, activation of GRB2 results in the upregulation of Sos protein 4. Sos protein activates Ras 5. Ras further triggers the stimulation of serine/threonine protein kinase Raf 6. Raf stimulates MEK1/2 7. MEK1/2 phosphorylates ERK1/2 (also known as MAPK) that regulates proliferation, differentiation and mitogenesis 8. Eventually the stimulation of MAP kinase signalling pathway together with the downregulation of PI 3-kinase pathway activate several molecular and cellular pathways that are linked to endothelial dysfunction, inflammation, increased oxidative stress and are significantly associated to the evolution of atherosclerosis. IR: Insulin resistance; PI 3-kinase: Phosphatidylinositol 3-kinase; IRS: Insulin receptor substrate; Sos: Son-of-sevenless; NO: Nitric oxide; MAP: Mitogen-activated protein; IR: Insulin resistance; HI: Hyperinsulinemia.

MAPK signalling cascades have critical effects on the transduction of extracellular messages to cellular activities. In mammalian cell lines, four MAPK cascades have been defined based on the components in the MAPK layer: (i) classical MAPK (also known as ERK1/2); (ii) JNK/ stress-activated protein kinase (JNK/SAPK) that promotes cell development, expansion, differentiation and survival; (iii) p38 MAPK, which is closely associated with inflammation, cell differentiation and proliferation; and (iv) ERK5 [73]. Tyrosine-phosphorylated Shc and IRS proteins bind to the SH2 domain of GRB2, promoting progressively the stimulation of the ERK/MAPK signalling pathway [62, 73-75]. In patients with elevated IR, cellular processes that promote the stimulation of the PI-3 kinase pathway are downregulated (selective IR). However, the ERK/MAPK signalling pathway stays insulin sensitive. Hence, it is excessively hyperactive. Stimulation of the ERK/MAPK pathway enhances the atherogenic and growth effects of insulin **(**Fig. **2**).

These detrimental activities are mainly achieved through (i) the activation of two key cellular signalling pathways, namely ΙΚΚβ/NF-κB and JNK. Macrophages and VSMCs, which were isolated both in animal and human atheromatous plaques, were shown to express prominently activated JNK signaling; (ii) the higher expression/secretion of endothelin 1 (ET-1), which is not only a potent vasoconstrictor but can also boost VSMCs growth and platelet adhesion. ET-1 receptors are also upregulated in VSMCs; (iii) higher prenylation of Ras and Rho (small molecular weight GTPases), which fosters stronger responsiveness of ECs to the atherogenic actions of VEGF and other growth molecules; (iv) stimulation of plasminogen activator inhibitor type 1 (PAI-1); (v) higher production of vascular cell adhesion molecules [such as E-selectin and vascular cell adhesion molecule 1 (VCAM-1)]; (vi) stronger interaction of ECs with rolling monocytes after VEGF activity; (vii) higher extracellular matrix protein expression; and (viii) suppressed proliferation and differentiation of endothelial progenitor cells (EPCs) [74-77].

Eventually, the stimulation of the ERK/MAPK signalling pathway together with the loss of NO bioactivity contributes to endothelial dysfunction. An endothelium with altered function is associated with a proinflammatory and prothrombotic state. Endothelial dysfunction: (i) stimulates the production of adhesion molecules; (ii) promotes macrophage penetration; (iii) stimulates the secretion of proinflammatory cytokines; (iv) enhances VSMCs growth and (v) favours platelet aggregation. A wealth of evidence has shown a strong connection between MAPK cascades with the uptake of oxidized LDL-C particles by macrophages and the promotion of foam cell formation. MAPK stimulation has been also intricately connected to the migratory/ proliferative ability of VSMCs and the development of neointima after vascular injury [73, 75-80].

## 4 HYPERINSULINEMIA AND ATHEROSCLEROSIS

Ample preclinical evidence (*in vitro* and *in vivo*) has demonstrated that high concentrations of insulin can promote atherosclerosis through several mechanisms: (i) stimulation of SREBP-1c, which has key roles in the activation glycolytic enzymes (especially glucokinase) and the expression of genes associated with lipogenesis. It stimulates acetyl-CoA carboxylase-1 (ACC-1) which is responsible for converting cytoplasmic acetyl-CoA into malonyl-CoA. In this way, it promotes *de novo* lipogenesis in hepatocytes from glucose. Hence, it increases hepatic fat content and promotes the production of *very-low-density lipoprotein cholesterol* (VLDL-C) in the circulation; (ii) growth and proliferation of VSMCs, ECs, and cultured preglomerular arteriolar smooth muscle cells; (iii) activation of collagen production and factors related to cell proliferation; (iv) upregulation of the expression/activity of LDL-C receptors; (v) stimulation of LDL-C transport into arterial smooth muscle cells and higher uptake of oxidized LDL-C particles by macrophages; (vi) promotion of lipid synthesis in the arterial wall; (vii) increased formation and decreased regression of lipid lesions [81-86]. Several preclinical models developed remarkable IR after insulin infusions for several days to maintain euglycemia; eventually, high BP and atherosclerosis were described [87, 88].

Furthermore, inappropriate initiation of insulin therapy in overweight/obese individuals with T2D should be carefully considered, bearing in mind the possible presence of IR syndrome (IRSyn) and its deleterious consequences. It must be emphasized that under normal conditions, approximately 66% of post-prandial insulin secretion is extracted from the portal system before it enters the peripheral circulation; it is used by the liver to promote glucose storage and to inhibit hepatic glucose production. The rest of the insulin penetrates the peripheral tissues. Administering exogenous insulin, cannot mimic neither the timing nor the magnitude of endogenous secreted insulin. It bypasses the human portal-systemic concentration gradient, which protects from peripheral hyperinsulinemia, while relatively higher insulin doses are needed to reach effective levels in the portal vein [89, 90]. Exogenous insulin is absorbed directly into the systemic circulation contributing to iatrogenic hyperinsulinemia, which exacerbates the pre-existing endogenous hyperinsulinemia and can stimulate several inflammatory and atherogenic signalling pathways [90, 91]. Indeed, Wang *et al* showed that iatrogenic hyperinsulinemia can stimulate pro-inflammatory macrophage responses associated with atherogenesis, both in mice and in patients with T1D [92]. Iatrogenic hyperinsulinemia can also stimulate lipogenesis, exacerbate cardiac IR, and promote endothelial dysfunction [90, 93]. Data from prospective follow-up studies demonstrated conflicting results as far as total insulin doses and higher risk of cardiovascular pathology. However, these trials were not organized to explore the question of whether increased insulin doses (daily doses and/or cumulative doses) were associated with higher cardiovascular risk and were characterized by several methodological flaws. Confounding by indication was the major problem since patients with higher IR, and thus increased probability for cardiovascular defects were given higher amounts of exogenous insulin. Insufficient information on exposure to higher insulin levels and several confounders, such as body mass index (BMI), duration of diabetes, and glycaemic control were other limitations reported in these studies [94].

In everyday clinical practice, large doses of daily exogenous insulin are necessary to achieve good glycaemic results in several individuals with T2D and high IR [usually ≥ 1 mg/kg daily to achieve glycated hemoglobin (A1C) levels less than 7%], which result in peripheral hyperinsulinemia and weight gain [95-97]. Patients who require more than 1 unit (U)/kg of daily exogenous insulin are considered to have IR, and those requiring more than 2 U/kg/day severe IR [98]. In a well-organized study published by Henry *et al,* it was reported that patients with T2D [mean BMI: 31.4±1.9 kg/m^2^, mean A1C: 7.7±0.4%], who were treated with insulin NPH and regular insulin for six months, experienced an average weight gain of 8.7 ± 1.9 kg *versus* pre-treatment values and peripheral hyperinsulinemia; increased body weigh was significantly associated to higher pre-treatment mean day-long serum insulin concentrations (*p<*0.05) and increased total exogenous insulin dose (*p<*0.02) [95].

**Fig. (3):**
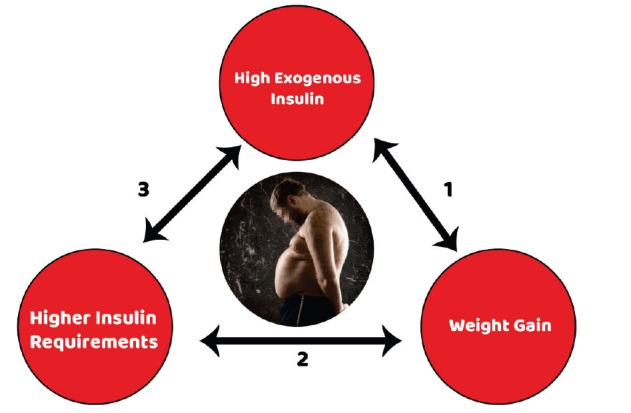
IRS and exogenous insulin therapy. **1.** IRS creates the need for more exogenous insulin requirements in patients with diabetes. High amounts of exogenous insulin promote weight gain through several mechanisms. **2.** Increased body weight exacerbates the preexisting IR state and subsequently leads to higher insulin requirements. **3.** Higher exogenous insulin doses contribute to iatrogenic hyperinsulinemia and can worsen the preexisting endogenous hyperinsulinemia, leading to a vicious-cycle of a feed-forward effect. IRS: Insulin resistance syndrome.

Body weight increases during the first 12 months of insulin initiation and is strongly associated with the insulin regimen and the intensity of treatment [99]. Higher body weight during the first six months after the initiation of insulin is mainly the result of reduced glycosuria and improved utilization of all metabolic substrates. Thereafter, it seems that other factors (independent of glycaemic control) are responsible for body weight gain and mainly: (i) anabolic effects of insulin to adipocytes and skeletal muscle cells; (ii) hypoglycaemia and defencing snacking; (iii) attenuation of the effects of insulin in promoting satiety; (iii) false sense of freedom to eat more food and depend on insulin to balance glucose concentrations; and (iv) multiple genetic factors [100, 101]. Increased body weight exacerbates IR, which in turn creates the need for more exogenous insulin requirements and subsequently worsens peripheral hyperinsulinemia leading to a vicious cycle of a feed-forward effect [101, 102] (Fig. **3**). An interesting study, which analyzed data from 192 patients with T2D for 12 months, showed approximately 3% body weight gain and about 23% higher insulin requirements during the first 12 months of insulin initiation both in individuals with normal and high baseline BMI levels [103]. A cross-sectional exploration of a representative sample of the NHANES database (2001 to 2010) suggested that patients with T2D and high IR (based on median HOMA-IR value > 4.55) were 2.45 times more likely to experience major adverse cardiovascular events (MACE) *versus* no-insulin users; this correlation remained meaningful after adjusting for several confounding factors (demographic characteristics, comorbid conditions, and A1C levels) [104]. No significant association was found in the low HOMA-IR arm. It was suggested that insulin administration may not always be beneficial in patients with T2D and high endogenous hyperinsulinemia, while for patients who experience predominantly an insulin secretory defect and lower IR levels, its use could be possibly more appropriate.

The cardiac and vascular safety, as well as the beneficial effects of insulin therapy, were shown in big pivotal randomized control trials (RCTs) of patients with T2D. However, mean insulin replacement doses in these studies were approaching the daily secretory values of individuals with NGT (insulin secretion in a lean individual with NGT is estimated near 0.2-0.5 U/kg). In the Outcome Reduction With Initial Glargine Intervention (ORIGIN) Trial, the median dose of insulin glargine increased from 0.31 U/kg to 0.40 U/kg by the sixth year [105]. In the DEVOTE (Trial Comparing Cardiovascular Safety of Insulin Degludec *versus* Insulin Glargine in Patients with Type 2 Diabetes at High Risk of Cardio-vascular Events) the median daily dose of insulin degludec ranged about 0.5 U/kg at 24 months, while in the landmark UK Prospective Diabetes Study (UKPDS) total daily insulin requirements were approximately 0.4 U/kg/day [106, 107]. Furthermore, in the pivotal DCCT study participants continued to gain body weight together with increases in insulin doses and waist circumference; 25% of intensively treated patients increased their BMI from a mean of 24 kg/m^2^ at baseline to at least 31 kg/m^2^ [18]. Excessive weight gain in the intensively treated arm (defined as at least an increase in BMI of 4.39 kg/m^2^) was sustained during 15 years of its observational follow-up study [Epidemiology of Diabetes Interventions and Complications (EDIC)]. It was related to worse BP and lipid control, increased coronary artery calcium values (a marker that has been significantly related to cardiovascular events and death over almost 15 years after screening at ages 38-55 years), and greater intima-media thickness *versus* minimal gainers [108-110]. Over 13 years of the EDIC study, the numbers of ASCVD events were not different between patients in the intensive insulin therapy arm who gained excessive weight *versus* those who gained small amounts of body weight (probably because of higher administration of lipid-lowering and blood pressure drugs in the excessive weight arm). However, after 14 years of the EDIC study, the event rate curves in the intensive arm started to diverge, with increased total cardiovascular event rates in the excessive weight gain arm *versus* the minimal weight gain arm, suggesting that non-traditional obesity associated ASCVD risk factors may promote contribution to the prevalence of atherosclerotic events in a delayed fashion. After 20 years of the EDIC study total ASCVD events in the excessive weight gainers in the insulin-intensive arm reached those found in the conventional treatment group of the DCCT [109]. Although the 30 years of follow-up in DCCT and EDIC showed that intensive insulin therapy decreased the incidence of any cardiovascular disease by 30% (*p=*0.016) and the incidence of major cardiovascular events by 32% (*p=*0.07), this must not minimize our efforts to limit excess weight gain that accompanies intensive insulin therapy in this population [111].

## 5 CARDIOMETABOLIC SYNDROME AND ATHEROSCLEROSIS

A wealth of evidence has connected the metabolic syndrome (the evolution of syndrome X that was discussed by Reaven in the historical 1988 Banting lecture) and/or IRSyn with ASCVD [112-115]. The cardiometabolic syndrome has been recognized as a disease entity by several scientific communities and organizations and has been closely associated with a cluster of abnormal conditions, which are independent risk factors for ASCVD (intra-abdominal obesity, glucose intolerance, hypertension, atherogenic dyslipidemia, and microalbuminuria); it is closely related to endothelial dysfunction, hyperuricemia, defective thrombolysis and increased oxidative stress/inflammation promoting the evolution of ASCVD [116, 117]. Approximately 25% of the adult population worldwide experience this syndrome. IR is its principal component and a key driver for its cardiovascular, metabolic, and renal sequelae [116-118]. The strong relationship between central obesity with both renal pathology and heart failure with preserved systolic activity has also led to the description of CardioRenal Metabolic Syndrome [119]. However, there is an unmet need to recognize IR as a major equivalent for cardiovascular disease (irrespective of any of the established criteria satisfied for the metabolic syndrome and/or for any of the definitions applied) and implement targeted therapeutic strategies to face it through its genesis [115, 118].

An important meta-analysis of 11 cohorts that were included in the European Diabetes Epidemiology Group, the Diabetes Epidemiology: Collaborative Analysis of Diagnostic Criteria in Europe (DECODE) study, explored evidence from 6156 men and 5351 women (30-89 years of age, average duration of follow-up: 8.8 years) [120]. The inclusion of two components of the metabolic syndrome (modified from the World Health Organization definition) showed 65.6% specificity for cardiovascular mortality in men, which increased to 85% if hyperinsulinemia was considered on top of them. In women, the inclusion of two components of the metabolic syndrome showed 71.6% specificity for cardiovascular mortality, which increased to 86.2% if hyperinsulinemia was calculated in the final analysis.

### 5.1 IR and Dyslipidemia

Long before individuals with IR experience abnormalities of glucose metabolism, high circulating levels of NEFAs are found. The main mechanism governing this phenomenon is the reduced activity of insulin to suppress and regulate lipolysis in adipocyte cells [121]. IR promotes increased VLDL-C levels through several mechanisms: (i) increased liver concentrations of NEFAs stabilize Apoprotein B-100 (apo B-100), the major core of VLDL-C. Higher hepatic concentrations of NEFAs are mainly the result of stimulated lipolysis from visceral fat (hormone-sensitive lipase cannot be sufficiently suppressed), *de novo* lipogenesis, and from the metabolism of intestinal chylomicrons; (ii) reduced degradation of Apo B-100, since the activity of the PI 3-kinase pathway is significantly downregulated; (iii) in states of IR the activity of FOXO1 cannot be suppressed. FOXO1 regulates the transcription of two major proteins: Apo C-III and microsomal triglyceride transfer protein (MTTP). Higher activity of FOXO1 leads to increased MMTP activity, the rate-limiting enzyme of VLDL-C production, and subsequently higher VLDL-C secretion. In addition, FOXO1 promotes higher transcriptional activity and hepatic secretion of ApoC-III, which inhibits lipoprotein lipase (LPL) in the endothelium of peripheral capillaries. LPL is the key mediator of VLDL-C clearance. It promotes hydrolysis and uptake of TG from chylomicrons and VLDL-C. Hence, it prolongs the persistence of VLDL-C in the circulation [122, 123].

Eventually, high levels of VLDL-C particles are metabolized to remnant lipoproteins and promote atheroma generation. Increased activities of cholesteryl ester transfer protein (CETP) and hepatic lipase are crucial for the production of small dense (sd) LDL-C particles through an indirect mechanism of increased rates of TG transfer from VLDL-C in exchange for cholesterol esters from LDL-C and *high-density lipoprotein* cholesterol (HDL-C) [124]. sdLDL-C can accumulate rapidly into the arterial wall since their smaller size can facilitate transendothelial transport and penetration to the subendothelial space. Moreover, sdLDL-C are vulnerable to several oxidative modifications, which subsequently increase their macrophages' capture and promote foam cell formation [124, 125]. Moreover, during an IR state the activity of CETP, which shifts TG from VLDL-C to HDL-C in exchange for cholesteryl esters, is stimulated. TG-enriched HDL-C particles undergo further modifications, including hydrolysis of their TG-part, leading to uncoupling of Apo A-I (which is cleared more rapidly from plasma). Hence, lower circulating ApoA-I levels and HDL-C particles are found. This contributes to lower HDL-C availability to participate in unloading cholesterol from the vasculature. HDL-C also has antithrombotic effects and antiatherogenic properties since it can stimulate eNOS and NO production [122, 124-126]. Interestingly, the AIP (a logarithmically transformed ratio of fasting TG to fasting HDL-C) has been associated with a higher risk of new onset stroke in both pre-diabetic and diabetic participants, while the TG and glucose index (TyG, a novel biomarker of IR) was found to be an independent risk factor for in-hospital death for patients with ST-segment elevation myocardial infarction (MI) and non-ST-segment elevation MI [127, 128]. TyG, as well as the systemic immune-inflammation index and systemic inflammation response index, have been associated with the evolution and severity of CAD in individuals with NAFLD [129].

### 5.2 IR and Hypertension

IR and compensatory hyperinsulinemia have been tightly connected to high BP through several mechanisms and mainly: (i) suppression of NO levels; (ii) increased ET-1 levels; (iii) higher sodium retention [higher sodium reabsorption after stimulation of Na-K-ATPase in the proximal convoluted tubule, as well as sodium proton exchanger type 3 (NHE3) stimulation]; (iv) stimulation of the sympathetic nervous system; (v) promotion of increased renal blood flow rates [since its direct vasodilatory effects to the pre-glomerular (afferent) arterioles are more intense *versus* those in the post-glomerular (efferent) arterioles], establishing hyperfiltration and microvascular remodelling; (vi) activation of renin-angiotensin aldosterone (RAAS) axis and stimulation of aldosterone production. Aldosterone is mainly produced from the zona glomerulosa in the adrenal cortex but it is also secreted from adipocytes (including perivascular adipocytes), and (vii) increased oxidative stress [130-133].

A meta-analysis exploring data from 11 prospective epidemiological trials suggested that the pooled relative risk of high blood pressure was 1.54 when comparing the biggest to the lowest group of fasting insulin levels, and 1.43 for comparing the biggest to lowest IR groups (as estimated by HOMA-IR) [134]. Furthermore, huge evidence suggests that IR has been associated with resistant hypertension, an independent risk factor for recurrent serious adverse cardiovascular episodes; obese individuals with resistant hypertension achieve excellent results when treated with mineralocorticoid receptor antagonists, if not contraindicated [135, 136]. Indeed, several molecules secreted from adipocytes can stimulate the adrenal secretion of aldosterone and cortisol [137, 138]. On the other hand, aldosterone can reduce insulin sensitiv ity through reduced GLUT-4 cell localization to the cellular surface, higher ROS production, and through serine/threonine phosphorylation of IRS1/2. Aldosterone can also boost the inflammatory phenotype of obesity and can indirectly enhance IR [139-141].

### 5.3 IR and Prediabetes

Prediabetes is an intermediate situation of glucose metabolism that exists between NGT and T2D and can be characterized as a continuous process from normal glucose levels to worsening glucose metabolism. It includes both impaired fasting glucose [(IFG)-fasting plasma glucose of 100 mg/dl to 125 mg/dl] and IGT [2-hour plasma glucose concentration of 140 mg/dl to 199 mg/dl after 75g oral glucose tolerance test (OGTT)]. Furthermore, A1C levels between 5.7% and 6.4% have also been suggested as another diagnostic criterion, although they were reported to predict poorly pancreatic β-cell dysfunction [142-144].

Both physical inactivity and the epidemic of obesity are IR states. When they are added to the genetic predisposition of IR they create huge stress on β-cells, which try to enhance their insulin secretion capacity to balance the defect of insulin activity [145]. Individuals with IGT have moderate to severe IR in the myocytes and normal to slightly reduced liver insulin sensitivity. They experienced abnormal patterns of insulin secretion, in both primary-phase (0-30 minutes) and late-phase (60-120 minutes), after OGTT. Individuals with IFG experience moderate IR in hepatic cells with normal insulin sensitivity in muscle cells and decreased basal and early phase of insulin release [142, 146]. Several epidemiological trials and meta-analyses have suggested that prediabetes (especially IGT) is strongly correlated to atherosclerosis and macrovascular complications [147-151]. Increased cardiovascular risk in individuals with IGT could be the result of several factors. Higher and longer daily glycaemic exposure of individuals with IGT, accompanied by greater fluctuations and variability, can stimulate several detrimental cellular pathways to the vasculature (increased inflammation, higher oxidative stress, defective coagulation, and abnormal vasomotion) *versus* individuals with IFG [144, 152-154]. Hyperglycaemia also stimulates the secretion of extracellular vesicles from several types of cells (such as ROS-producing and LDL-C scavenging CD36), which promote atherosclerosis through specific proteins [155].

### 5.4 IR, Obesity, Inflammation, Oxidative Stress and Hypercoagulation

A wealth of information during the last four decades has been accumulated regarding the formation and activity of white adipose tissue, which has substantially clarified our knowledge of the pathophysiology of IR [156]. Adipose tissue in obesity is signalized by adipocyte hypertrophy, enhanced angiogenesis, extracellular matrix overload, and immune cell penetration [157, 158]. The enlargement of adipose tissue is tightly related to higher macrophage infiltration of the “classically activated” M1 phenotype, which is related to a pro-inflammatory state, compared to the “alternative activated” M2 phenotype shown in lean adiposity [158, 159]. Both adipose tissue cells and macrophages produce various adipocytokines and inhibit others (such as adiponectin). In this way they: (i) establish a multiplex network of molecules and signaling pathways, which stimulate, maintain and exacerbate an IR state; and (ii) stimulate several aberrant cellular signalling pathways (mainly JNK and IKKβ/NF-kB), which are associated with the production of key stimulating inflammatory mediators that promote inflammation, oxidative stress, endothelial dysfunction and eventually atherogenesis [156-158]. Moreover, it has been suggested that leptin resistance and/or impaired leptin secretion relative to fat mass could stimulate the generation of ectopic visceral adipose tissue, which is involved in the pathogenesis of atherosclerosis [160].

Furthermore, accumulating evidence has shown a close association between abdominal obesity/IR and hypercoagulation through reduced fibrinolysis, higher thrombin production, and increased platelet activity [161]. This prothrombotic state is chiefly the result of several proinflammatory adipokines (such as MCP-1 and TNF-α) that stimulate prothrombotic molecules in adipocytes of obese individuals [162, 163]. Specifically, higher procoagulant effects of circulating monocyte tissue factor are found, as well as increased plasma concentrations of coagulation factor VIIa (FVII) and PAI-1. Both thrombin and thrombin-antithrombin complexes are also upregulated [161-164]. Moreover, preclinical and clinical data have demonstrated resistance to the anticoagulant activity of antiplatelet therapy in these states [164].

## 6 THERAPEUTIC PROSPECTS

Lifestyle modifications (improved dietary habits and enhanced physical exercise) are the cornerstones for the management of obesity and IR and they demand stable and durable adjustment on a life-long schedule regardless of whether other treatment options are implemented [165]. A 5% decrease in body weight was shown to enhance insulin sensitivity by almost 30% and reduce the progression of IGT to T2D by approximately 58%, in the Diabetes Prevention Program [166, 167]. Unfortunately, significant and constant weight loss with only lifestyle changes, in individuals with obesity and IR with or without T2D, remains difficult and challenging [102, 168]. Bariatric surgery can be a successful and generally secure procedure for the treatment of severe obesity and promotes extended weight reduction and improvement/suppression of several obesity-associated comorbid diseases and syndromes (such as T2D, OSA, and PCOS) [169-171]. However, a recent review, which analyzed data from 7391 Roux-en-Y gastric bypass and 5872 sleeve gastrectomy individuals, suggested that at least 1 in 6 patients after bariatric surgery experienced 10% or more weight gain [172]. A wealth of evidence also suggests that inflammatory responses and immunoregulation are key players in the evolution of atherosclerosis, in all plaque formation events (from the beginning of the plaque until the loss of stability inside the fibrous cap and its rupture); IR was shown to be associated with necrotic plaque volume and vulnerable plaque composition in asymptomatic men without diabetes, although its exact role needs further investigation in this setting [8, 173-176]. Interestingly, it was very recently shown that both the constancy and the monocyte phenotypic characteristics were tightly associated with cardiovascular events in individuals with T2D [177]. Moreover, when data from 31,245 patients treated with contemporary statins were recently analyzed, it was shown that high-sensitivity CRP (hs-CRP) was a more powerful predictor for future cardiovascular episodes and death *versus* LDL-C cholesterol levels [178]. Hence, combining non-pharmaceutical strategies with pharmaceutical agents with broader specificity and novelty is crucially needed to face drastically IR and its sequelae in their genesis [179].

During the last two decades, evidence from patients with active autoimmune diseases and high IR states has suggested that anti-TNF therapy can enhance insulin sensitivity and can reverse several abnormal processes in the insulin signaling pathway [180, 181]. Anti-TNF therapies achieved the highest decreases in CRP and *erythrocyte sedimentation rate (*ESR), which could not be justified by alterations in body structure, suggesting that by suppressing inflammation, IR can be reduced [181]. Interesting results were also reported when anti-TNF therapies were administered in states of IR and metabolic syndrome, while a recent large meta-analysis investigating anti-inflammatory therapeutic approaches *versus* placebo in individuals with severe cardiovascular danger or with documented ASCVD suggested that anti-inflammatory therapeutic strategies (mainly aiming the IL-6 pathway) may become useful therapeutic approaches to reduce the probability of experiencing myocardial infarction MI [182-184]. Colchicine 0.5 mg per day achieved a significantly lower percentage of ischemic cardiovascular events *versus* placebo among individuals with recent MI [185]. It was found to exert anti-atherosclerotic and plaque-stabilizing activities by suppressing foam cell formation and inflammation from cholesterol crystals [186]. It also significantly improved several obesity-related inflammatory indices and markers of IR in obese individuals, who experience metabolic syndrome without T2D [187].

A recent *post hoc* exploratory analysis of data from the Insulin Resistance Intervention after Stroke (IRIS) study, which explored the effect of pioglitazone (PIO) dose regarding its efficacy and tolerability in a high IR population, suggested that even 15 mg of PIO given every day can decrease the probability of experiencing stroke/MI by 47% having a good safety profile [188]. PIO can reduce IR not only in adipocytes, hepatocytes, and skeletal myocytes but also in cells that have direct effects on the evolution of atherosclerosis such as ECs and VSMCs [4, 158, 189, 190]. Furthermore, replacing insulin administration after combining liraglutide with duodenal mucosal resurfacing (a minimally invasive endoscopic technique that ablates duodenal mucosa and promotes future regeneration) for six months succeeded discontinuation of insulin in 69% of patients with T2D who were enrolled and improved total metabolic and glycaemic health; modulation of postprandial bile acid response was shown to result in changes of the microbiome, ileal bile acid balance and eventually enhanced insulin sensitivity [191, 192]. Indeed, restoring and modifying gut microbiota can play an interesting role in host metabolism and the evolution of IR; ongoing studies are starting to unravel the cellular events, that play critical roles in this setting [193, 194]. Low concentrations of indole-3-propionic acid (IPA), a tryptophan catabolic product mainly produced by C. Sporogenes, have been associated with IR and low-grade inflammation, suggesting that IPA could be a useful tool to improve metabolic homeostasis in humans [195].

Several phase I-III studies have been launched to investigate novel drugs that can suppress hepatic *de novo* lipogenesis and stimulate NEFA oxidation in skeletal muscles [196]. Mitochondria uncouplers, thyromimetics, fibroblast growth factor analogs, and nicotinamide adenine dinucleotide precursors are also under intense investigation to suppress IR [197]. Peptide derived from PKC Alpha Targeting AlmS (PATAS) is the first-in-class peptide that aimed adipocytes (ALMS1- PKCa protein interaction) and promoted several metabolic chain events that suppressed IR and related comorbid conditions *in vivo* [198]. Furthermore, mounting preclinical and clinical evidence suggests that genetic alterations in certain genes (such as *NAT2*, and *SLC16A11*) can lead to reduced mitochondrial function and promote liver and muscle IR. Exploring the exact molecular mechanisms governing their effects will be crucial to developing targeted therapies for suppressing IR [199, 200]. Dysregulation of microRNAs (miRNAs) has been also recently shown to exert important effects in the induction of IR through targeting several signalling pathways related to glucose homeostasis and lipid metabolism. miRNAs can be beneficial therapeutic targets since they can alter the expression of several related target genes, and future research is needed [201]. Ferroptosis, a novel cell death defined by a huge buildup of lipid peroxides due to intracellular iron storage, has been linked to the pathophysiology of several diseases, including obesity, T2D, and atherosclerosis; targeted approaches of ferroptosis related to these diseases are crucially anticipated [202].

## CONCLUSION

Chronic IR isn’t a disease but preferably a definition of a physiological condition, in which any individual has a higher probability of experiencing closely associated metabolic abnormalities and clinical syndromes, as well as the development of future vascular resistance and arterial stiffness [203-205]. Almost every IR state is characterized by a dysfunctional endothelium, which is now considered as the pivotal key event in the evolution of atherosclerosis [206, 207]. Evidence from the Framingham studies suggested that overweight and obese individuals significantly experience ASCVD (after correction for traditional risk factors) [208]. Indeed, several studies have raised the speculation that traditional risk factors for ASCVD can justify approximately 70% of observed cardiovascular events and that most of the unaccounted risk could be explained by IR/hyperinsulinemia *per se* [208-210]. Hence, beyond the current approach to treat aggressively all classical cardiovascular risk factors there are unmet clinical demands to be answered. Understanding the contribution of IR/hyperinsulinemia to the pathophysiology of atherosclerosis is of immense importance to visualize and develop novel therapeutic strategies and targets for treatment, beyond lifestyle modifications and surgical anti-obesity interventions [4, 146, 196, 210, 211].

## Figures and Tables

**Table 1:** Insulin effects on main target cells.

**Target Cell**	**Main Effects**	**References**
*Skeletal muscle cell*	(i) Glucose transport, through stimulation and translocation of GLUT-4 to the cell surface; (ii) glucose phosphorylation through hexokinase-II stimulation; (iii) glycogen synthesis *via* the activation of GYS. It also controls glycogen phosphorylase activity through dephosphorylation of phosphorylase kinase; and (iv) stimulation of mTORC1, which suppresses autophagy and regulates protein synthesis and cell growth.	[31-33]
*Hepatic cell*	(i) Suppression of hepatic glucose production, namely glycogenolysis and gluconeogenesis. The suppression of gluconeogenesis is achieved by inhibiting lipolysis from the adipose tissue (thus reducing glycerol substrates) and by Akt-induced phosphorylation of FOXO1 through IRS-2; (ii) enhances glycogen synthesis after the regulation of GYS and glycogen phosphorylase; and (iii) stimulates *de novo* lipid anabolism by activating its key transcriptional regulator SREBP-1c through IRS-1.	[34-38]
*Adipocyte*	(i) Suppression of lipolysis, which in turn downregulates gluconeogenesis through decreased gluconeogenic substrates. This effect is mainly achieved by attenuating c-AMP stimulated adrenergic activities through PDE3B activation; and (ii) promotes lipogenesis after: (α) regulating FATPs and NEFAs esterification; (β) by activating SREBP-1c; and (γ) by regulating the expression of PPAR-γ in human adipocytes.	[39-42]
*VSMC and EC*	VSMCs and ECs express insulin receptors. Phosphorylation of insulin receptors causes tyrosine phosphorylation of IRS-1, IRS-2 and Shc. In this way both PI 3-kinase and ERK/MAPK pathways are activated in a normal metabolic state. Furthermore, through the stimulation of PI 3-kinase/Akt pathway (mainly through IRS-1) insulin promotes the phosphorylation of eNOS, stimulating its expression and activity. Eventually it increases NO levels that promote smooth muscle relaxation and vasodilation.	[41-44]
*Monocyte/* *Macrophage*	Insulin signaling is also activated in monocytes/macrophages, although IRS-1 isoform is indetectable. Suppression of the insulin pathway in monocytes was shown to decrease the protective activity of insulin to suppress macrophage apoptosis.	[43, 45, 46]
*Other cells*	In renal cells insulin plays an important role to the modulation of gluconeogenesis and lipolysis. In cardiomyocytes, activation of the insulin pathway regulates diverse cellular processes including cell growth and survival and suppression of apoptosis and autophagy. Activation of insulin signaling in brain cells controls nutrient homeostasis, cognition and several important neurotrophic and neuromodulatory activities.	[47-49]

**Note:** GLUT-4: Glucose transporter 4; GYS: Glycogen synthase; FOXO1: Forkhead box O1; IRS: insulin receptor substrate; m-TOR: Mammalian target of rapamycin; SREBP-1c: Sterol regulatory element-binding protein 1c; PDE3B: Phosphodiesterase 3B; FATPs: Fatty acid transport proteins; NEFAs: Non-esterified fatty acids; PPAR-γ: Peroxisome proliferator-activated receptor-gamma; VSMC: Vascular smooth muscle cell; EC: Endothelial cell; ERK/MAPK: Extracellular signal-regulated kinase/MAPK: *Mitogen*-activated protein kinase; PI 3-kinase: Phosphatidylinositol 3-kinase; eNOS: endothelial nitric oxide synthase; Ref.: Reference.

**Table 2:** Main molecules/signaling pathways that interfere with the insulin pathway and suppress the activity of PI 3-kinase pathway.

**Molecules/Pathways**	**Main Activities**	**References**
Adipocytokines (such as TNF-α, IL-6)	Suppression expression of insulin receptor, IRS-1 and GLUT-4. Promotion of serine-phosphorylation of IRS-1/2.	[4, 53, 51-53]
m-TOR/S6K1	m-TOR/S6K1 kinase activation during hyperinsulinemia and overnutrition promotes serine phosphorylation of IRS-1. Hence, disruption of IRS-1 interaction with the insulin receptor and inhibition of PI3K-mediated Akt activation are found.	[50, 51-53]
JNK and IKKβ /NF-kB	JNK and IKKβ/NF-kB promote IRS serine phosphorylation after the arrival of several extracellular signals (such as adipocytokines and lipid intermediates).	[4, 50, 56, 65, 66]
PKC isoforms	Abnormal expression and activation of PKC isoforms promote serine /threonine phosphorylation of IRS.	[4, 56, 194]
Lipid intermediate metabolites (LPA, DAG, acylcarnitines, ceramides)	DAG is a key molecule in this setting and inhibits the insulin signalling pathway by increasing IRS serine/threonine phosphorylation. Ceramides can suppress PI-3 kinase/Akt pathway activation through: (i) increased protein phosphatase-2A activity that leads to dephosphorylation and inactivation of Akt; and (ii) after impairing Akt translocation to the plasma.	[55, 55, 57, 58]
Fatty acyl-CoA	They stimulate IKKβ/NF-kB signalling pathway and promote the secretion of major stimulating key-inflammatory mediators (TNFα, ILs and PKC).	[55, 57, 58, 194]
ROS	High ROS levels can promote IR by affecting several key proteins in the insulin pathway (such as IRS-1 phosphorylation/redistribution).	[56, 59, 61, 62, 65, 66]
Ang II	It can promote IR though ROS-mediated serine phosphorylation of IRS-1 on VSMCs and skeletal muscle cells and though JNK/MAPK/ERK1/2 serine phosphorylation of IRS-1 in the ECs. Mineralocorticoid receptors activation also stimulate the mTOR/S6K1 signaling pathway that promotes IRS-1 serine phosphorylation in adipocytes, hepatic cells, skeletal muscle cells and cardiac cells.	[63, 64]

**Note:** IR: Insulin resistance; IRS: insulin receptor substrate: GLUT: Glucose transporter; PI 3-kinase: Phosphatidylinositol 3-kinase; m-TOR: Mammalian target of rapamycin; JNK: c-Jun N-terminal kinase; IKKβ: IkB kinase β; NF-kB: Nuclear factor-kβ; LPA: Lysophosphatidic acid; DAG: Diacylglycerol; PKC: Protein kinase C; IL: Interleukins; TNFα: Τumor necrosis factor α; ROS: Reactive oxygen species; MAPK: *Mitogen*-activated protein kinase; ERK: Extracellular signal-regulated kinase; Ang II: Angiotensin II; VSMCs: Vascular smooth muscle cells; Ref.: Reference.

## LIST OF ABBREVIATIONS

ASCVD: Atherosclerotic Cardiovascular Disease
ECs: Endothelial Cells
HIEC: *Hyperinsulinemic-Euglycemic* Clamp
IGT: Impaired Glucose Tolerance
LDL-C: Low-Density Lipoprotein Cholesterol
MAPK: Mitogen-Activated Protein Kinase
NGT: Normal Glucose Tolerance
PCOS: Polycystic Ovary Syndrome
RCTs: Randomized Control Trials
T2D: Type 2 Diabetes
